# Epistasis: Searching for Interacting Genetic Variants Using Crosses

**DOI:** 10.1534/genetics.117.203059

**Published:** 2017-06-06

**Authors:** Ian M. Ehrenreich

**Affiliations:** Molecular and Computational Biology Section, Department of Biological Sciences, University of Southern California, Los Angeles, California 90089-2910

**Keywords:** epistasis, genetic interactions, linkage mapping, crosses, multiparental populations (MPP)

## Epistasis Matters in Quantitative Genetics

Within quantitative genetics, the term “epistasis” is used to broadly describe situations in which combinations of genetic variants show nonadditive phenotypic effects (Phillips 1998, 2008; Mackay 2014). Although most work on epistasis has focused on pairs of variants that interact (Brem *et al.* 2005; Bloom *et al.* 2015), more complicated forms of epistasis can also occur (Taylor and Ehrenreich 2015a). These include higher-order interactions between three or more variants (Rowe *et al.* 2008; Pettersson *et al.* 2011; Taylor and Ehrenreich 2014) and cases in which one variant acts as a hub of interactions with a number of other variants (Carlborg *et al.* 2006; Forsberg *et al.* 2017).

Despite many reports of epistasis, its importance to quantitative genetics remains under active debate (Huang and Mackay 2016). This is in part because theory suggests that even if epistasis is present, most genetic variance will be additive (Hill *et al.* 2008; Maki-Tanila and Hill 2014). Consistent with this argument, purely additive models explain most of the heritability of many quantitative traits (Bloom *et al.* 2013) and have proven quite effective in crop and livestock breeding programs (Crow 2010). Given that epistasis can be ignored to little detriment, what do we gain by studying epistasis?

Epistasis matters for multiple reasons. A central goal of quantitative genetics is to determine the genetic architectures that underlie heritable traits (Mackay 2001). By definition, this endeavor entails identifying nearly all of the genetic effects that appreciably influence phenotypes, including epistatic effects. Achieving such a precise understanding of genotype–phenotype relationships advances our basic knowledge of genetics and may improve our ability to predict traits, such as disease risk and crop yield, from genome sequences (Forsberg *et al.* 2017). Because epistasis often reflects functional relationships between genes, finding interacting variants can also shed light on molecular mechanisms that give rise to trait variability (Aylor and Zeng 2008; Rowe *et al.* 2008; Cordell 2009; Huang *et al.* 2012; Taylor *et al.* 2016).

Furthermore, epistasis impacts our understanding of why genetically distinct individuals respond differently to new spontaneous and induced mutations (Nadeau 2001; Queitsch *et al.* 2002; Mackay 2014; Siegal and Leu 2014; Schell *et al.* 2016). Such background effects are common across species and traits, and are known to contribute to clinically relevant phenotypes (Nadeau 2001; Chandler *et al.* 2013). Recent work has shown that genetic background effects often reflect complex interactions between new mutations and multiple segregating variants (Dowell *et al.* 2010; Chari and Dworkin 2013; Chandler *et al.* 2014; Paaby *et al.* 2015; Taylor and Ehrenreich 2015b; Geiler-Samerotte *et al.* 2016; Lee *et al.* 2016; Taylor *et al.* 2016). Thus, predicting how individuals will respond to new mutations, including genetic changes introduced by genome editing (Cong *et al.* 2013; Mali *et al.* 2013), will likely require accounting for epistasis.

## Challenges in Using Genetic Mapping to Detect Epistasis

Identifying epistasis is difficult because most genetic mapping studies are only capable of detecting the simplest and largest-effect interactions (Taylor and Ehrenreich 2015a). Although selective genotyping approaches can be used to find interacting variants (Ehrenreich *et al.* 2010; Taylor and Ehrenreich 2014, 2015b; Lee *et al.* 2016; Taylor *et al.* 2016), usually epistasis is identified by association or linkage mapping (Marchini *et al.* 2005; Cordell 2009; Verhoeven *et al.* 2010; Bloom *et al.* 2015; Forsberg *et al.* 2017).

A common challenge in genome-wide scans for epistasis is multiple testing (Cordell 2009; Sham and Purcell 2014). The number of tests in a scan for epistasis will scale almost exponentially with the order of the interactions being considered (Cordell 2009). For example, assuming the number of variants in a population equals 10,000, then the number of tests in genome-wide scans for pairwise, three-way, and four-way epistasis will be ∼5×10^7^, ∼2×10^11^, and ∼4×10^14^, respectively. With these large numbers of tests, stringent statistical approaches must be employed to minimize false positives (Sham and Purcell 2014).

A related difficulty that genome-wide scans for epistasis face is statistical power. Leveraging data from multiple traits (Tyler *et al.* 2013, 2017), searching for epistatic effects involving variants that also have additive effects (Storey *et al.* 2005; Laurie *et al.* 2014), jointly modeling additive and epistatic effects (Marchini *et al.* 2005; Verhoeven *et al.* 2010), and identifying variants that respond to genetic background (Jannink and Jansen 2001) or show effects on phenotypic variance (Ronnegard and Valdar 2011) are just some of the approaches that can aid in the detection of interacting variants. Yet arguably the best solution to the statistical power problem is to use very large sample sizes in genome-wide scans for epistasis (Bloom *et al.* 2013, 2015; Hallin *et al.* 2016). Notably, both overall sample size in a study and sample sizes within multilocus genotype classes must be considered (Carlborg and Haley 2004). Sample sizes within multi-locus genotype classes should ideally be balanced, but in some cases this may not be possible, for example when association mapping is performed on natural isolates that possess population structure and a spectrum of allele frequencies (Mackay *et al.* 2009).

Another factor that may be important to detecting epistasis is how often the involved variants also show additive effects. This question has bearing on whether efforts to identify epistasis can be simplified into a two-step process in which additive variants are first identified and then their interactions are measured. Recent work indicates that interacting variants also tend to exhibit additive effects (Bloom *et al.* 2015). However, in some cases, new mutations appear to interact with “cryptic” variants that do not typically influence phenotype (Gibson and Dworkin 2004; Paaby and Rockman 2014), suggesting that major epistatic effects can involve variants that lack additive effects.

## Exploring Epistasis with Crosses

One of the best opportunities for identifying interacting variants is using linkage mapping in crosses of genetically diverse isolates from model species (Carlborg and Haley 2004; Mackay *et al.* 2009; Taylor and Ehrenreich 2015a). In many of these organisms, isolates can be made homozygous by inbreeding [*e.g.*, *Drosophila* (Mackay *et al.* 2012) and mouse (Beck *et al.* 2000)], sporulation [*e.g.*, budding yeast (Liti *et al.* 2009; Schacherer *et al.* 2009)], or creation of doubled haploids [*e.g.*, many plants (Maluszynski *et al.* 2003)], enabling the generation of stable genotypes that minimize heterozygosity. Using inbred lines as the founders of crosses is desirable because it allows unambiguous cataloging of the variants that will segregate among progeny. Recombinant inbred lines (RILs) can then be produced from cross progeny in the same way that the inbred founders were generated (Carlborg and Haley 2004; Mackay *et al.* 2009; Taylor and Ehrenreich 2015a).

RILs represent a powerful resource for identifying epistatic effects because they carry random combinations of the variants that differentiate their founders and have minimal to no population structure (Carlborg and Haley 2004; Rockman 2008; Mackay *et al.* 2009; Taylor and Ehrenreich 2015a). There are many experimental design choices to make when constructing RIL populations (Verhoeven *et al.* 2006; Rockman and Kruglyak 2008; Mackay *et al.* 2009). Assuming sample size is not limiting, one of the key decisions in constructing a cross is the number of founders (Kover *et al.* 2009; Aylor *et al.* 2011; Long *et al.* 2014). While two-parent RIL populations are commonly used, multi-parent RILs can be generated from dozens of founders or more (Ladejobi *et al.* 2016).

As highlighted by the rapidly growing “Multiparental Populations” series in *GENETICS* and *G3: Genes│Genomes│Genetics* (de Koning and McIntyre 2014), there is tremendous interest in using RIL populations derived from more than two founders to examine the genetic basis of quantitative traits. A number of crossing designs have been described for generating multiparent RILs. These include, but are not limited to, employing multiple rounds of crossing to ensure that each founder contributes equally to each RIL (Churchill *et al.* 2004), nested association mapping (NAM) in which one common founder is crossed to many others (McMullen *et al.* 2009), and crossing each founder to two or more of the other founders in a full or partial diallel design (Verhoeven *et al.* 2006; Treusch *et al.* 2015). Multiparent RILs can also be interbred to produce outbred populations that resemble natural populations but lack population structure (Svenson *et al.* 2012). Relative to more traditional two-parent crosses, multiparent populations have some clear advantages: they sample a greater fraction of the genetic diversity that exists within a species and can lead to finer mapping resolution (Yu *et al.* 2008; Kover *et al.* 2009; Aylor *et al.* 2011; Long *et al.* 2014).

## Trade-Offs in Searching for Epistasis Using Multiparent Crosses

Regarding epistasis, the main strength of multiparent populations relative to two-parent crosses is a more complete sampling of the combinations of interacting variants that segregate in a species. However, the specific crossing design used to generate multiparent RILs will influence the epistatic effects that are detectable. For example, the maize NAM population was generated by mating 25 genetically diverse founders to the same reference line (B73) and producing RILs from each two-parent cross (Yu *et al.* 2008; Buckler *et al.* 2009; McMullen *et al.* 2009). The NAM panel provides a compelling opportunity to identify interactions involving variants carried by B73 (Yu *et al.* 2008; Peiffer *et al.* 2014). However, this population might have more limited potential to identify other epistatic effects.

Generating multiparent RILs that are equally derived from each founder can maximize the epistatic effects present in a cross, but has consequences for multi-locus genotype frequencies at interacting variants. While two-parent RILs have the advantage that all variants and two-locus combinations should segregate at ∼1/2 and ∼1/4, respectively, this is not the case for multiparent RILs. For example, the eight founders of the mouse Collaborative Cross contribute almost equally to each RIL (Churchill *et al.* 2004; Aylor *et al.* 2011; Collaborative Cross Consortium 2012), implying that minor allele frequencies should be between ∼1/8 and ∼1/2 among the RILs. This variability in allele frequencies can lead to low and unbalanced multi-locus genotype frequencies at interacting variants, which may result in false negatives in genome-wide scans for epistasis. In an extreme case where two founder-specific variants interact, each will occur in roughly an eighth of the RILs and the four multi-locus genotype frequencies involving the variants will have frequencies of ∼1/64, ∼7/64, ∼7/64, and ∼49/64. Despite this issue, multiparent populations like the Collaborative Cross can be a very useful resource for studying epistasis, especially when systems-level data are available or information is leveraged across traits (Tyler *et al.* 2017).

An additional factor to consider when using multiparent populations to study epistasis is allelic heterogeneity, which occurs when multiple causal variants reside in either the same gene or different, closely-linked genes (Risch 2000; Long *et al.* 2014; Matsui *et al.* 2015; Linder *et al.* 2016). Many cases of allelic heterogeneity have been found in both multiparent genetic mapping (Buckler *et al.* 2009; Ehrenreich *et al.* 2012; King *et al.* 2012, 2014; Peiffer *et al.* 2014) and association studies (Lango Allen *et al.* 2010; Hormozdiari *et al.* 2016). With respect to epistasis, this allelic heterogeneity may make it more difficult to detect interacting variants in multiparent populations than in comparably sized two-parent populations.

## Conclusion

Epistasis has important phenotypic effects, but can be difficult to identify. RILs produced by crossing genetically distinct isolates can facilitate the detection of interacting variants, but experimental design criteria must be considered, including how many founders to employ. Expanding the genetic variation that is present in a cross by using more founders has both advantages and disadvantages. For example, RILs produced by crossing two founders will have balanced multilocus genotype frequencies, which can provide statistical power to identify pairwise and higher-order epistasis. However, comprehensively mapping epistatic effects across a species requires using a number of founders. These considerations speak to how epistasis is a complex and incompletely understood phenomenon that has no single form. Thus, assuming finite resources, the most appropriate experimental design for studying epistasis may depend on the specific question one wants to address.
